# Seasonal Impact on Wound Healing and Surgical Site Infections after Reduction Mammoplasty

**DOI:** 10.3390/jcm13195938

**Published:** 2024-10-05

**Authors:** Maximilian Mahrhofer, Glenda Giorgia Caputo, Frederic Fierdel, Raphael Reichert, Elisabeth Russe, Florian Wimmer, Thomas Schoeller, Laurenz Weitgasser

**Affiliations:** 1 Department of Plastic and Reconstructive Surgery, Marienhospital Stuttgart, Teaching Hospital of the Eberhard Karls University, 70199 Tuebingen, Germany; 2 Clinic of Plastic and Reconstructive Surgery, Ospedale Santa Maria della Misericordia, 33100 Udine, Italy; sblenda@yahoo.it; 3 Department of Plastic and Reconstructive Surgery, Hospital of the Brothers of St. John of God, Paracelsus Medical University Salzburg, 5010 Salzburg, Austria

**Keywords:** mammoplasty, breast reduction, seasonal impact, surgical site infection, wound healing

## Abstract

**Background:** The incidence of reduction mammoplasty has been steadily increasing over recent decades. Surgical site infections (SSIs) represent a common yet preventable complication across surgical disciplines. Studies across various surgical specialties have indicated a seasonal influence on SSIs, primarily correlated with higher temperatures and humidity. However, there remains a scarcity of clear data regarding the seasonal effects on complications specifically in breast surgery. **Methods:** We conducted a retrospective review encompassing all patients who underwent primary bilateral reduction mammoplasties at our institution between 1 June 2016, and 1 September 2019. The data collected included patient demographics, surgical details, and postoperative complications. The rates of SSIs and wound healing disturbances (WHDs) were correlated with local meteorological data at the time of surgery. **Results:** A total of 808 patients (1616 breasts) met the inclusion criteria. The mean age was 41 ± 14.8 years, with a mean BMI of 28.9 ± 5.2 kg/m^2^ and a mean follow-up duration of 8.9 ± 9.8 months. Nineteen cases (2.35%) of surgical site infections and 77 cases (9.52%) of wound healing disturbances were reported. No statistically significant increase in the risk of SSIs (*p* = 0.928) or WHDs (*p* = 0.078) was observed during the warmer months of the year. Although no specific risk factors were identified for surgical site infections, both resection weight (*p* < 0.001) and diabetes mellitus (*p* = 0.001) demonstrated increased risks for wound healing disturbances. **Conclusions:** While seasonal temperature variations have been shown to impact SSIs and WHDs in body contouring procedures, our findings suggest that breast reduction surgery may not be similarly affected.

## 1. Introduction

The rapid proliferation of online information and the increasing prevalence of overweight and obesity have led to a significant surge in demand for reduction mammoplasty in recent decades [1,2]. This surgical procedure greatly enhances patients’ overall well-being and quality of life [3,4]. Although it is generally regarded as safe, the medical literature frequently reports complication rates that range from 20% to 50% [5,6]. Most of these complications are minor, such as wound healing disturbances (WHDs) and fat necrosis. However, surgical site infections (SSIs), although often preventable, remain prevalent and significantly contribute to the overall healthcare-related costs associated with infections [7].

While major SSIs following reduction mammoplasty are relatively rare, they do pose substantial health risks to patients and may necessitate re-hospitalization and revision surgery, potentially affecting esthetic outcomes. This situation can be particularly challenging for reduction mammoplasties performed in outpatient or private settings. Given that cost coverage by health insurance varies widely, this financial risk for patients is often overlooked [8]. A multitude of risk factors for WHDs and SSIs have been documented in the medical literature, including smoking, diabetes, body mass index (BMI), and the duration of the operative procedure. Previous studies have investigated the impact of seasonal temperature variations on infection rates and other complications that arise after surgery. Gruskay et al. demonstrated higher rates of infections following spinal surgeries in the United States during the end of summer and the beginning of fall [9]. Conversely, in their extensive study involving over 100,000 patients after total hip arthroplasty in the United States, Ng et al. did not find any significant seasonal changes in complications, except for an unexplained risk of bleeding [10]. Similar findings without increased complication rates were reported for trauma-related injuries in the Netherlands [11]. Beyond postoperative surgical complications, seasonal variations for other issues, such as thromboembolic events, have also been described, but a uniform consensus on this remains elusive [12,13,14].

While the influence of temperature and humidity on SSIs and WHDs has been thoroughly explored in the fields of orthopedics and neurosurgery, there is scant evidence regarding its impact within plastic surgery [15,16,17,18]. Duscher et al. demonstrated a significant increase in SSIs during warmer months by analyzing a diverse cohort of body contouring procedures, which included 194 patients undergoing breast reduction surgeries [19]. Similarly, an elevated risk of WHDs during warmer seasons was noted in carpal tunnel surgery, and higher complication rates during summer months were observed in reconstructive surgeries that utilized free flaps for breast and extremity reconstruction [20,21]. With global temperatures on the rise and record-breaking heatwaves occurring annually, the environmental impact on surgical complications warrants serious consideration and further investigation.

This study aims to evaluate the influence of seasonal changes in temperature and air humidity on WHDs and SSIs following reduction mammoplasty, involving a large cohort of 808 patients over a four-year period at a single center.

## 2. Materials and Methods

A retrospective review of surgical records was conducted, including all adult patients who underwent primary bilateral reduction mammoplasties between 1 June 2016, and 1 September 2019. The review involved a comprehensive examination of relevant medical records, including submission charts and surgical notes, to gather essential patient data. Both patients with symptomatic breast hypertrophy and those undergoing the procedure for cosmetic purposes were included in the analysis. Available patient demographics and risk factors (age, body mass index (BMI), smoking, and diabetes mellitus) were collected. Additionally, detailed surgical information was recorded, including operating time, type of skin incision used, resection weight, and whether perioperative antibiotic prophylaxis was administered. Electronic records were screened for postoperative WHDs and SSIs within the first 30 days after surgery. Complications that were diagnosed both during the inpatient hospital stay and after discharge were included in the analysis. All surgeries were performed by a team of surgeons with varying experience levels, including resident-led procedures supervised by board-certified surgeons. Patients received perioperative antibiotic prophylaxis, but standard postoperative antibiotic treatment was not administered. One drain per breast was used for all patients and usually removed between postoperative days 1–4, when the output reached < 20 mL/24 h. The planned discharge for all patients was set at two days following surgery. The study was performed in accordance with the Declaration of Helsinki and the Guidelines for Good Clinical Practice. The safety of patient data and the protection of privacy were ensured. Institutional review board approval was obtained prior to commencement. 

Official weather data for the study period (1 June 2016, to 1 September 2019) were sourced from the department of environmental protection by the city of Stuttgart, Germany, (Amt für Umweltschutz, Abteilung Stadtklima). In order to account for seasonal weather variations, monthly temperature and air humidity levels were analyzed and correlated with postoperative complication rates. Patients were divided into two cohorts based on annual temperature and monthly sunlight exposure in the northern hemisphere. All surgeries performed between 1 June and 31 August were registered in the “warm” season while surgeries between 1 September and 31 May were registered in the “cold” season. Additionally, groups were formed based on the meteorological temperature seasons of the northern hemisphere: summer (June, July, August), autumn (September, October, November), winter (December, January, February), and spring (March, April, May). While all surgeries were performed in standardized climate-controlled operating rooms, no external climate control devices, such as air conditioners, were used in the patient rooms postoperatively during their hospital stay.

Wound healing disturbance was defined as any superficial or deep-tissue healing problem larger than 2 mm within 90 days after surgery. The diagnosis of SSIs was made clinically during planned or emergency follow-ups, in any case showing signs of infection such as fatigue, fever, local or spread inflammation signs, pus, or secretion of the wound. Treatment consisted of conservative treatment, oral or intravenous antibiotics and surgical intervention. 

Infection rates and wound healing problems were calculated as a percentage of the total surgeries performed during each season/period. Infection rates were then compared using the chi-square test, with a *p* value less than 0.05 considered significant.

Continuous variables are expressed as means with standard deviations (SD). Univariate statistical analyses included independent two-sided t-tests for categorical variables and binominal logistic regression for metric data. Odds ratios were calculated for risk factors and reported with their 95% confidence interval (95% CI). Statistical analysis was performed using Microsoft Excel (v16.89.1 Microsoft Corp., Redmond, WA, USA) and R Statistical Software (v4.3.2 R Core Team 2023).

## 3. Results

A total of 808 patients (1616 breasts) met the inclusion criteria. Mean age was 41 ± 14.8 years, mean BMI 28.9 ± 5.2 kg/m^2^ and mean follow-up 8.9 ± 9.8 months (Table 1). Twenty-three patients (2.8%) had a history of diabetes mellitus and 176 (21.8%) were active smokers. A Wise pattern skin incision was used in all 1616 breasts and the mean operative time was 129 ± 30.1 min. The superomedial pedicle (41.6%) and the inferior pedicle (34.5%) were most commonly used. Mean resection weight was 636.8 ± 316.2 g and the majority of patients (95.5%) had their surgery covered by their health insurance.

Average temperatures during the study period ranged from 3.7 ± 3.9 °C (January) to 22.1 ± 0.7 °C (July), while average humidity was inversely correlated with a maximum of 77.4 ± 1.19% in December and the lowest percentage in April (56.0 ± 6.08%) (Table 2).

**Figure 1:**
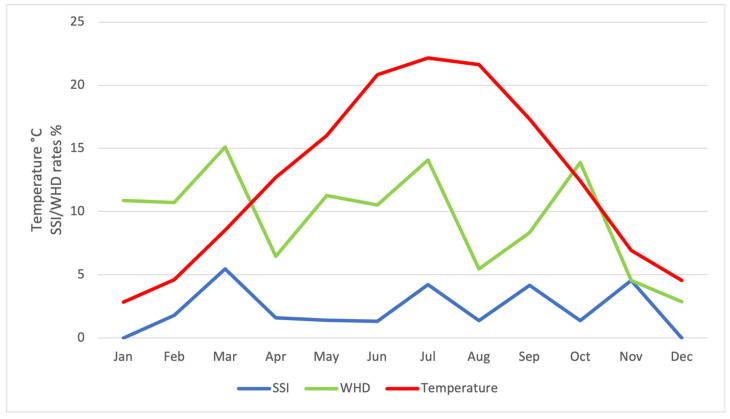
Local temperature curve (°C) for the study period. Surgical site infection (SSI) and wound healing disturbance (WHD) rates are added. No statistical significance was shown.

**Figure 2:**
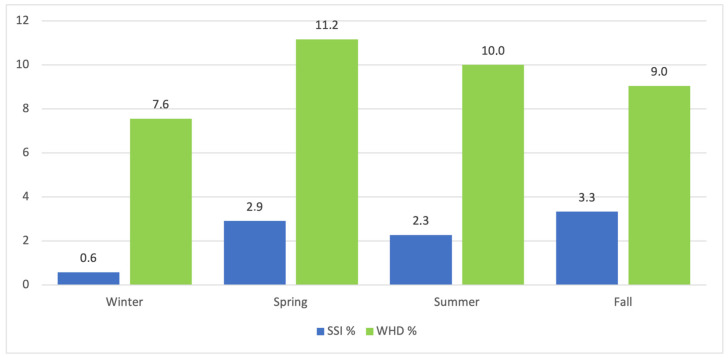
Relative occurrence rates of surgical site infections (SSIs) and wound healing disturbances (WHDs) grouped by season.

**Figure 3:**
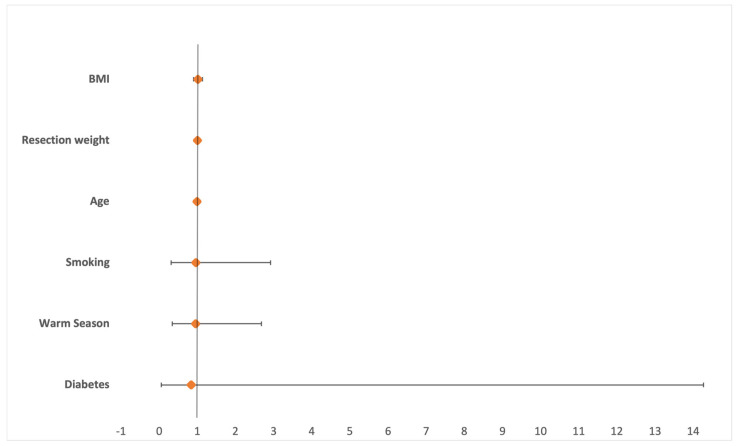
Logistic regression analysis for the occurrence of surgical site infections. None of the analyzed risk factors reached statistical significance (BMI = body mass index).

**Figure 4:**
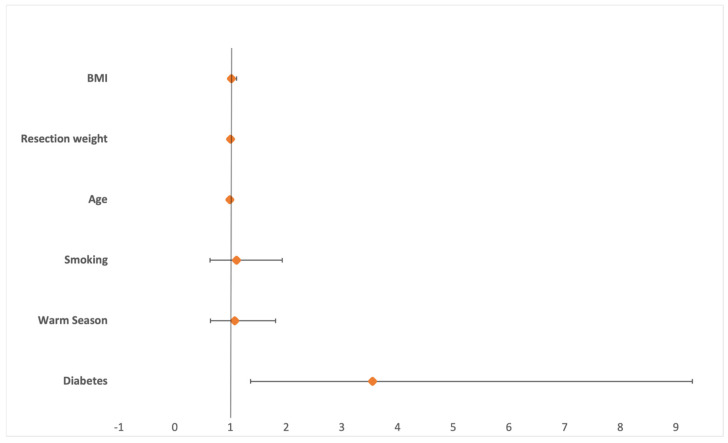
Logistic regression analysis for the occurrence of wound healing disturbances. Diabetes (OR 3.549; *p* = 0.001) and resection weight per g (OR 1.001; *p* < 0.001) were shown to significantly increase the risk of WHDs (BMI = body mass index).

Nineteen (2.35%) surgical site infections and 77 (9.52%) wound healing disturbances were reported. The highest rates of SSI were found in March (5.5%) and November (4.5%) whereas wound healing disturbances most commonly occurred in March (15.1%) and July (14.1%). While winter showed the lowest number of SSIs (0.6%) and WHDs (2.9%), wound healing disturbances were most frequent in spring (11.2%) and SSIs in fall (3.3%) (Figure 1). Statistical analysis showed no increased risk for SSIs (OR 0.954 [95%CI 0.34–2.68]), *p* = 0.928) or WHD (OR 1.077 [95%CI 0.64–1.81]; *p* = 0.078) in the warm period of the year (Figure 2 and Figure 3). While no risk factors were identified for surgical site infections; resection weight (OR 1.001 [95%CI 1.000–1.001]; *p* < 0.001) and diabetes mellitus (OR 3.549 [95%CI 1.36–9.29]; *p* = 0.001) both demonstrated increased risks for wound healing disturbances (Table 3, Figure 4). 

## 4. Discussion

Patients seeking consultation for elective plastic surgery are often hesitant to schedule their procedures during the warmer months of the year, driven by the widespread belief that elevated temperatures increase the risk of wound infections. Although this assumption seems logical, there is little scientific evidence to substantiate or refute this belief. Postoperative outcome studies constitute a significant portion of the available surgical literature; however, the limitation of these studies is that they only analyze the risk factors that researchers specifically choose to include. Consequently, most outcome studies tend to focus on major risk factors, such as pre-existing health conditions, surgical techniques, age, or smoking habits [22]. As a result, there is limited scientific research available regarding the influence of external factors, such as weather, on postoperative complications. This lack of evidence is particularly relevant in the context of elective procedures, which are common in plastic surgery. Patients undergoing elective surgeries often exhibit heightened skepticism and concern about potential complications. These fears and uncertainties must be addressed preoperatively by surgeons, especially in an age where unchecked digital information and social media can perpetuate misconceptions. One common concern patients voice preoperatively is the fear of increased wound healing issues and infections during warmer seasons, yet little concrete scientific data exists to support surgeons in alleviating these fears. Our study does not support the widespread assumption that warmer weather increases the risk of surgical site infections (SSIs) or wound healing disturbances (WHDs) following breast reduction surgery. Although this hypothesis, which has been previously posited by other researchers, seems reasonable—given the extensive wound surfaces and complex scar characteristics of body contouring procedures—our findings diverge from this expectation. Earlier studies have indeed identified seasonal variations in postoperative infection risks, including certain plastic surgery procedures [18,20,23,24,25,26,27]. For instance, Duscher et al. observed a significant rise in risk for body contouring surgeries during the warmer months [19]. However, a key difference lies in the patient demographics and their underlying health conditions. Body contouring surgeries, such as thigh lifts or abdominoplasties, are often performed following significant weight loss, whether achieved through surgery or major lifestyle changes. In contrast, patients seeking reduction mammoplasties generally have a history of attempts at weight loss aimed at reducing breast size, but typically not to the same degree as those undergoing body contouring.

Although perioperative antibiotic prophylaxis guidelines in plastic surgery are not comprehensive, they have been widely adopted and were consistently utilized in our study cases [28]. For example, a survey of Brazilian plastic surgeons found that 75% of respondents prescribed antibiotics for the postoperative period following breast reduction surgery [28]. Seretis et al. found a significant reduction in postoperative SSIs after reduction mammoplasty, while showing no effect on delayed or prolonged wound healing [29]. While individual patient risks, such as underlying comorbidities or local medical resources, may vary, our study indicates that SSIs after reduction mammoplasty are generally rare. On the other hand, wound healing disturbances and prolonged wound healing are more frequently observed, consistent with the findings of previous research [6,30,31].

In their 2022 review of 10 studies, Froschauer et al. demonstrated a seasonal risk increase for WHDs in carpal tunnel surgery (OR 1.07, *p* = 0.002), yet our data do not support a similar conclusion for reduction mammoplasties [21]. In contrast, Garoosi et al. identified a notable increase in the risk of postoperative complications, including wound dehiscence and SSIs, after body contouring surgeries. Their study included a significant number of breast surgeries and identified risk factors such as diabetes, obesity, tobacco use, and hypertension [32]. While Malone et al. previously demonstrated an association between diabetes and SSIs, our study did not corroborate this finding. However, diabetes was found to significantly increase the risk of wound healing problems (OR 3.549, *p* = 0.001) [33]. Furthermore, it is important to recognize that social vulnerability and socioeconomic factors can also influence postoperative outcomes and the risk of wound infections [34]. Previous studies have also mentioned a possible influence of seasonal staff changes, such as the influx of new hires or interns during the summer, on increased complications [9]. Although our study did not reveal any seasonal patterns of increased complications, we acknowledge that this factor might contribute to higher complication rates, as shown in earlier research. A study by Henson et al., including over 200,000 spinal surgery patients from a national data base, found slightly higher risks for urinary tract infections (UTIs) and hospital readmission after surgery during the warm season [17].

Resection weight plays a pivotal role in reduction mammoplasty and other body contouring procedures. De Kerviler et al. demonstrated an increased risk of postoperative complications associated with higher resection weights following body contouring in massive weight loss patients [35]. Similarly, in breast reduction surgery, this correlation has been established in prior studies and was confirmed by our data to increase the likelihood of wound healing complications [5,36].

In their review, Leehka et al. identified a seasonal variation in Staphylococcus aureus skin and soft tissue infections, with peak incidences occurring during the summer and fall months [37]. However, the included studies showed significant variability in terms of geographic region, infection type (such as tissue infection, bacteremia, or colonization), and study design. Geographic and socioeconomic factors are likely to be closely related to postoperative infections. For instance, while Kaier et al. did not find a clear association between temperature and S. aureus infections in two German hospitals, Elegbe et al. demonstrated a temperature-related seasonal effect on coagulase-positive Staphylococci boil infections in Nigerian patients [38,39].

### Limitations

Our study has several limitations, including its retrospective design, which may introduce inherent weaknesses such as data collection errors or missing data. Retrospective studies rely on pre-existing data, which may be incomplete, inconsistent, or inaccurately recorded. This can introduce errors and limit the reliability of the findings. Additionally, our data were derived from a single study site in western Europe, where moderately mild summer temperatures prevail; thus, the observed effects of seasonal temperature may not be generalizable to regions with more extreme climates. Furthermore, operations were performed by multiple surgeons with varying levels of experience, including plastic surgery residents. Differences in experience or technical choices may potentially influence the outcome to an unknown extent. Although all patients were routinely invited for clinical follow-ups and encouraged to seek treatment for surgical complications, cases treated outside our institution may not have been captured. While patients are encouraged to attend their postoperative follow-up, complications such as wound healing disturbances or infections might be treated outside the hospital due to geographical distance, socioeconomic, or other reasons. This adds a potential selection bias to our study. Detailed socioeconomic data are missing and could be a potential confounder. Additionally, our inclusion criteria relied on clinical diagnosis of SSIs rather than microbiological or blood testing. Finally, daily weather variations and other factors such as patient hygiene, clothing, or individual wound care may potentially influence complication rates.

## 5. Conclusions

This study showed no increased risk for surgical site infections or wound healing problems during the warm months of the year at a western European hospital. While a potential effect was shown for body contouring procedures in general, this may not be applicable for reduction mammoplasties, due to the smaller scars, better reachability for wound care and hygiene, as well as different tissue composition. Diabetes and resection weight need to be considered as important risk factors for postoperative wound healing problems.

## Figures and Tables

**Table 1:** Patient Characteristics and Operative Details.

	Overall	Warm Season	Cold Season
**Characteristic**	**No. (%)**	**No. (%)**	**No. (%)**
Patients	808 (100)	220 (27.2)	588 (72.8)
Breasts	1616 (100)	440 (27.2)	1176 (72.8)
Mean age ± SD, years	41.1 ± 14.8	41.0 ± 15.5	41.0 ± 14.5
Mean BMI ± SD, kg/m^2^	28.9 ± 5.2	29.0 ± 5.3	28.9 ± 5.1
Diabetes mellitus	23 (2.8)	6 (2.7)	17 (2.9)
Smoking	176 (21.8)	50 (22.7)	126 (21.4)
Mean follow-up ± SD, months	8.9 ± 9.8	9.0 ± 10.1	8.8 ± 9.6
Insurance coverage	772 (95.5)	207 (94.1)	565 (96.1)
**Operative Details**			
Wise pattern	1616 (100)	440 (100)	1176 (100)
Pedicle			
Superomedial	673 (41.6)	186 (42.3)	487 (41.4)
Inferior	557 (34.5)	144 (32.7)	413 (35.1)
Superolateral	301 (18.6)	79 (17.9)	222 (18.9)
Superior	85 (5.3)	31 (6.8)	54 (4.6)
Mean resection weight ± SD, g	636.8 ± 316.2	663.9 ± 331.1	626.6 ± 309.8
Mean operative time ± SD, min	129 ± 30.1	143 ± 31.3	124 ± 28.1

BMI = body mass index, SD = standard deviation

**Table 2:** Monthly Overview of Weather and Complications.

	Avg. Temperature	Avg. Humidity	No. of Patients	SSI (%)	WHD (%)
Month	in °C	in %	n = 808	n = 19	n = 77
January	3.7 ± 3.9	75.6 ± 1.28	46	0	5 (10.9)
February	5.5 ± 2.7	67.3 ± 3.27	56	1 (1.8)	6 (10.7)
March	8.0 ± 1.8	63.3 ± 3.02	73	4 (5.5)	11 (15.1)
April	12.5 ± 2.4	56.0 ± 6.08	62	1 (1.6)	4 (6.5)
May	15.7 ± 1.8	61.6 ± 3.27	71	1 (1.4)	8 (11.3)
June	20.4 ± 1.6	61.5 ± 4.78	76	1 (1.3)	8 (10.5)
July	22.1 ± 0.7	56.4 ± 4.17	71	3 (4.2)	10 (14.1)
August	21.8 ± 0.9	61.1 ± 4.04	73	1 (1.4)	4 (5.5)
September	17.4 ± 1.6	65.5 ± 4.07	72	3 (4.2)	6 (8.3)
October	12.7 ± 1.3	73.1 ± 2.30	72	1 (1.4)	10 (13.9)
November	7.3 ± 0.8	76.5 ± 1.86	66	3 (4.5)	3 (4.5)
December	5.0 ± 1.1	77.4 ± 1.19	70	0	2 (2.9)

SSI = surgical site infection, WHD = wound healing disturbance > 2 mm.

**Table 3:** Univariate Risk Factor Analysis for Complications.

	SSI		WHD	
Variable	Odds Ratio (95% CI)	*p*	Odds Ratio (95% CI)	*p*
Age, years	0.988 (0.95–1.02)	0.472	0.988 (0.98–1.01)	0.129
BMI, kg/m^2^	1.014 (0.90–1.13)	0.811	1.014 (0.99–1.11)	0.735
Warm season	0.954 (0.34–2.68)	0.928	1.077 (0.64–1.81)	0.078
Diabetes	0.836 (0.05–14.27)	0.902	3.549 (1.36–9.29)	0.001
Smoking	0.957 (0.31–2.92)	0.938	1.107 (0.63–1.93)	0.722
Resection weight, g	1.000 (0.99–1.00)	0.423	1.001 (1.000–1.001)	<0.001

SSI = surgical site infection; WHD = wound healing disturbance > 2 mm; CI = confidence interval; BMI = body mass index.

## Data Availability

Available upon reasonable request.
